# Online detection and quantification of epidemics

**DOI:** 10.1186/1472-6947-7-29

**Published:** 2007-10-15

**Authors:** Camille Pelat, Pierre-Yves Boëlle, Benjamin J Cowling, Fabrice Carrat, Antoine Flahault, Séverine Ansart, Alain-Jacques Valleron

**Affiliations:** 1 Université Pierre et Marie Curie-Paris 6, UMR-S 707, Paris, 75012 France; 2 INSERM, U707, Paris, 75012 France; 3 AP-HP, Hôpital Saint Antoine, Unité de Santé Publique, Paris, 75012 France; 4 Department of Community Medicine and School of Public Health, The University of Hong Kong, Hong Kong SAR, China; 5 AP-HP, Hôpital Tenon, Unité de Santé Publique, Paris, 75012 France; 6 Hôpital de la Cavale Blanche, Maladies infectieuses, Brest, 29609 cedex, France

## Abstract

**Background:**

Time series data are increasingly available in health care, especially for the purpose of disease surveillance. The analysis of such data has long used periodic regression models to detect outbreaks and estimate epidemic burdens. However, implementation of the method may be difficult due to lack of statistical expertise. No dedicated tool is available to perform and guide analyses.

**Results:**

We developed an online computer application allowing analysis of epidemiologic time series. The system is available online at . The data is assumed to consist of a periodic baseline level and irregularly occurring epidemics. The program allows estimating the periodic baseline level and associated upper forecast limit. The latter defines a threshold for epidemic detection. The burden of an epidemic is defined as the cumulated signal in excess of the baseline estimate. The user is guided through the necessary choices for analysis. We illustrate the usage of the online epidemic analysis tool with two examples: the retrospective detection and quantification of excess pneumonia and influenza (P&I) mortality, and the prospective surveillance of gastrointestinal disease (diarrhoea).

**Conclusion:**

The online application allows easy detection of special events in an epidemiologic time series and quantification of excess mortality/morbidity as a change from baseline. It should be a valuable tool for field and public health practitioners.

## Background

The generalization of electronic data capture in health care has made time series data increasingly available for public health surveillance [1]. How to best analyse these data will likely be case dependent and require expert statistical advice. There is however a well agreed "good analysis practice" in particular classes of surveillance problems, so that less expert users may consider undertaking the analysis themselves. This requires making software available online and providing guidance on its use: this is exactly what was done with online tools for DNA sequences alignment (BLAST, FASTA), allowing biologists to successfully use these methods on their own data.

Here, we focus on epidemic detection and quantification from time series data. There is a widely used approach for this purpose originating from Serfling's work on influenza [2]. He proposed calculating excess P&I mortality due to seasonal influenza using deviations from a periodic regression model that captured the annual seasonality of the data. It was first necessary to (subjectively) select years without excess death to train the baseline regression model. The approach has then been extended to address several issues: refining regression equations and extracting baseline model information without subjective filtering of the data [3-5]. Algorithms for prospective outbreak detection were also proposed in this framework [6-8].

In this paper, we describe an online tool allowing users to detect unexpected events, eg outbreaks, in a seasonal epidemiologic time series. Two applications are detailed to illustrate how results are obtained.

## Implementation

Two types of analysis exist for surveillance time series: retrospective analysis, to locate and quantify the impact of past epidemics, and prospective analysis, for real time detection of epidemics. In all cases, four steps are necessary. First, a subset of data ("training data") is selected from the whole time series to estimate the baseline level. Second, an algorithm or a rule is used to selectively discard epidemic events from the training data, so that the baseline level is estimated from truly non epidemic data. Third, a periodic regression model is fitted to the training data. Finally, the model is used to define an epidemic threshold and/or estimate excess morbidity/mortality. We review how these issues have been addressed in the literature, using the detection of influenza epidemics in time series as an illustration. Table 1 summarizes all inputs required from the user, and describes the default options retained in our system.

**Table 1:** Required inputs from the user for baseline model fitting

**Parameter**	**Possible values**	**Default value**
Length of the training period	Number of years, number of observations	Retrospective : All data Prospective : Half dataset
Purge of the training period	Data above selected percentile, above cut-off value, or in user-defined periods	Above the 15% highest percentile
Regression equation	Linear, quadratic or cubic terms. Annual, semi-annual or quarterly periodicity	Automated model selection
Upper forecast limit (UFL)	Percentile between 50% and 100%	95%
Minimum duration above UFL defining an unexpected change	Number of observations	14 days/2 weeks/1 month

### Training period

Even if long time series are available, it is not generally the case that all data should be included in the training period [9]. Indeed, changes in case reporting and demographics will likely be present over long time periods, and this may affect how well the baseline model fits the data. Modelling of influenza mortality typically uses the five preceding years in baseline determination [2,10,11]. Including more past seasons improves the seasonal components estimates, while limiting the quantity of data allows capturing recent trends. In our system, we propose using the whole dataset in the model fitting for retrospective analysis (as done, for example, in [12,13]), and to limit to a past few years in the case of prospective detection of epidemics (as, for example, in [7,8]). In the latter case, the user is invited to specify the length of the training data in an input field. He can define it in number of years or in number of observations. In either case, the minimal time span accepted is one year.

### Purge of the training period

In order to model the non-epidemic baseline level, the model must be fitted on non-epidemic data. For seasonal diseases such as influenza in the Northern hemisphere, it is difficult to find long epidemic-free periods since epidemics typically occur every year. There are two choices to deal with the presence of epidemics in the training data: excluding the corresponding data from the series, or explicitly modelling the epidemics.

In the first choice, epidemics must first be identified. Several rules have been suggested in this respect. Viboud et al. excluded the 25% highest values from the training period [13]. Costagliola et al. removed all data above a given threshold (more than three influenza-like illness cases per sentinel general practitioner) [14]. Olson et al. excluded the months with "reported increased respiratory disease activity or a major mortality event" [4]. Others deleted entire periods: e.g. December to April [12], or September to mid-April [15].

The second choice, less common, requires explicit modelling of the epidemic periods during the training data. In this case, an epidemic indicator must be included as a covariable in the model. For influenza epidemic, one may choose the number of laboratory influenza A and B isolates [5,16]. However, the availability of an independent epidemic indicator is uncommon in practice.

In summary, data points may be excluded either because they exceed a (possibly data determined) threshold, because they were collected during a period known to be epidemic prone (for example winters), or because the user wishes to exclude the points. These three options are available in our system.

### Regression equation

A variety of formulations may be used for the regression equation, including linear regression [14], linear regression on the log-transformed series [6], Poisson regression [17], and Poisson regression allowing for over-dispersion [18]. Linear regression is suitable when working with large frequencies or incidences, while working with the log transformed series or applying Poisson regression is advised when observations are small in magnitude.

In the regression equation, the trend is generally modelled using a linear term [2,4,11], or a second degree polynomial [3,7,19]. In our application we propose these two trends plus the third degree polynomial, to offer more flexibility. When the model is used for prospective detection of epidemics, it is often safer to use only a linear trend to avoid inconsistencies when the model will be extrapolated into the future. Thus, the application restrains the user's choice to the models that have linear trend. For retrospective analysis, where extrapolation is not an issue, more complex trends may improve the fit of the baseline model. So, the application allows the user to choose among all the proposed models with linear, quadratic and cubic trends. For the seasonal component, a simple yet effective description may be obtained using sine and cosine terms with period one year [2]. Refined models are found in the literature, often with terms of period 6 months [14], sometimes 3 months [3], and, rarely, smaller [11]. In our application, we chose to propose the most widely used periodicities, ie 12, 6 and 3 months. As a result, all regression equations for the observed value Y(t) are special cases of the following model: Y(t) = α_0 _+ α_1 _t + α_2 _t^2 ^+ α_3 _t^3 ^+ γ_1 _cos(2πt/n) + δ_1 _sin(2πt/n) +γ_2 _cos(4πt/n) + δ_2 _sin(4πt/n) + γ_3 _cos(8πt/n) + δ_3 _sin(8πt/n) + ε(t). For prospective modelling, α_2 _and α_3 _are always 0. Model coefficients are estimated by least squares regression.

**Figure 1:**
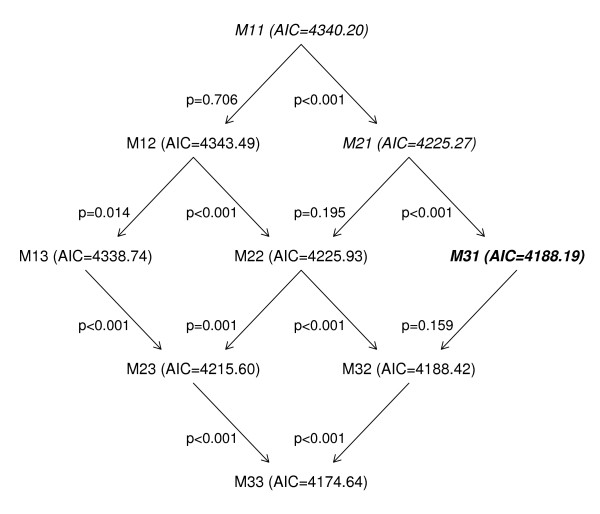
**Model selection algorithm**. Graphical output of the model selection algorithm. Data and models are described in Table 2. Models selected through the algorithm pathway are in italics. The model finally kept is in bold italics.

In our system, automatic selection of the best fitting model is made possible by a selection algorithm (see Figure 1, which illustrates the process on an example detailed in the result section). It relies on ANOVA comparison (significance level : 0.05) to select between nested models, and on Akaike's Criterion to select between non-nested models [20]. The algorithm starts comparing, by ANOVA, the simplest model M11 (Y(t) = α_0 _+ α_1 _t + γ_1 _cos(2πt/n) + δ_1 _sin(2πt/n) + ε(t)) with the two models in which it is nested: M12 (Y(t) = α_0 _+ α_1 _t + γ_1 _cos(2πt/n) + δ_1 _sin(2πt/n) + γ_2 _cos(4πt/n) + δ_2 _sin(4πt/n) + ε(t)) and M21 (Y(t) = α_0 _+ α_1 _t + α_2 _t^2 ^+ γ_1 _cos(2πt/n) + δ_1 _sin(2πt/n) + ε(t)). If none of the alternative models (M12 and M21) is significantly better than the initial one (M11), the algorithm keeps M11 and stops. If one of the two alternative models is better than the initial one, the algorithm keeps it and goes on. If the two alternative models are better than the initial one, the algorithm keeps the one with the lowest AIC and goes on. The process is repeated until finding the "best overall" model over the nine proposed models.

**Table 2:** Retrospective evaluation of the excess P&I mortality in France for 1968–1999, using nine periodic regression models. The components included in each model are indicated by a*. ^#^Model options: exclusion of the top 15% percentile from the training period; forecast interval: 95%

	**Trend**			**Periodicity**				
**Model**^#^	**t**	**t^2^**	**T**^3^	**1 year**	**6 months**	**3 months**	**AIC**	**Cumulated excess mortality over the whole period**

**M11**	*			*			4 340	88 442
**M12**	*			*	*		4 343	87 260
**M13**	*			*	*	*	4 339	88 266
**M21**	*	*		*			4 225	85 083
**M22**	*	*		*	*		4 226	83 245
**M23**	*	*		*	*	*	4 216	83 505
**M31**	*	*	*	*			4 188	85 175
**M32**	*	*	*	*	*		4 188	83 337
**M33**	*	*	*	*	*	*	4 175	82 465

### Alert notification

As the baseline model is fitted to the observations, the variation around the model fit may be estimated by the standard deviation of the residuals (difference between observed and model value). It is therefore possible to calculate forecast intervals for future observations, assuming that the baseline model holds in the future. Thresholds signalling an unexpected change are typically obtained by taking an upper percentile for the prediction distribution (assumed to be normal), typically the upper 95th percentile [14], or upper 90th percentile [11]. A rule is then used to define when epidemic alerts are produced: for example as soon as an observation exceeds the threshold [7], or if a series of observations fall above the threshold, for example during 2 weeks [13], or 1 month [21].

## Results

We developed a web-based application allowing users to construct periodic regression models for analysis of epidemiologic time series. It is written in HTML, PHP, and JavaScript for the user interface, and interfaced with the R system (2.5.0) for statistical computations [22]. The application is available online [23]. The R codes are freely available in Additional file 1 and on the application web site.

Users may input their own dataset (eg incidences, mortalities, medication sales) as a plain text file (ie ASCII file) containing the time series as a single column, i.e. the values are separated by a carriage return. Observations must be aggregated by day, week or month. The user will be invited to specify this time step in a scrolling list. Missing values are allowed, provided they are coded by "NA". It is assumed that the dataset will contain at least one year of data. Several example datasets from France are included in the system: incidence rates per 100,000 population for influenza-like illness and diarrhoea for 1991–2001, and P&I mortality series for 1968–1999 [24]. They are available as daily, weekly or monthly time series.

### Retrospective analysis of influenza epidemics

The first example uses monthly P&I mortality in France over the period 1968–1999. The user wishes to retrospectively identify the epidemic periods and quantify the cumulated mortality in these epidemics. Use of the system begins with selecting the corresponding dataset on the main page.

After data input, the user is taken through three successive webpages to specify the baseline model parameters (Table 1). The first page allows choosing the type of analysis. Here, the user selected to conduct retrospective analysis, therefore the whole time series is included in the training period.

**Figure 2:**
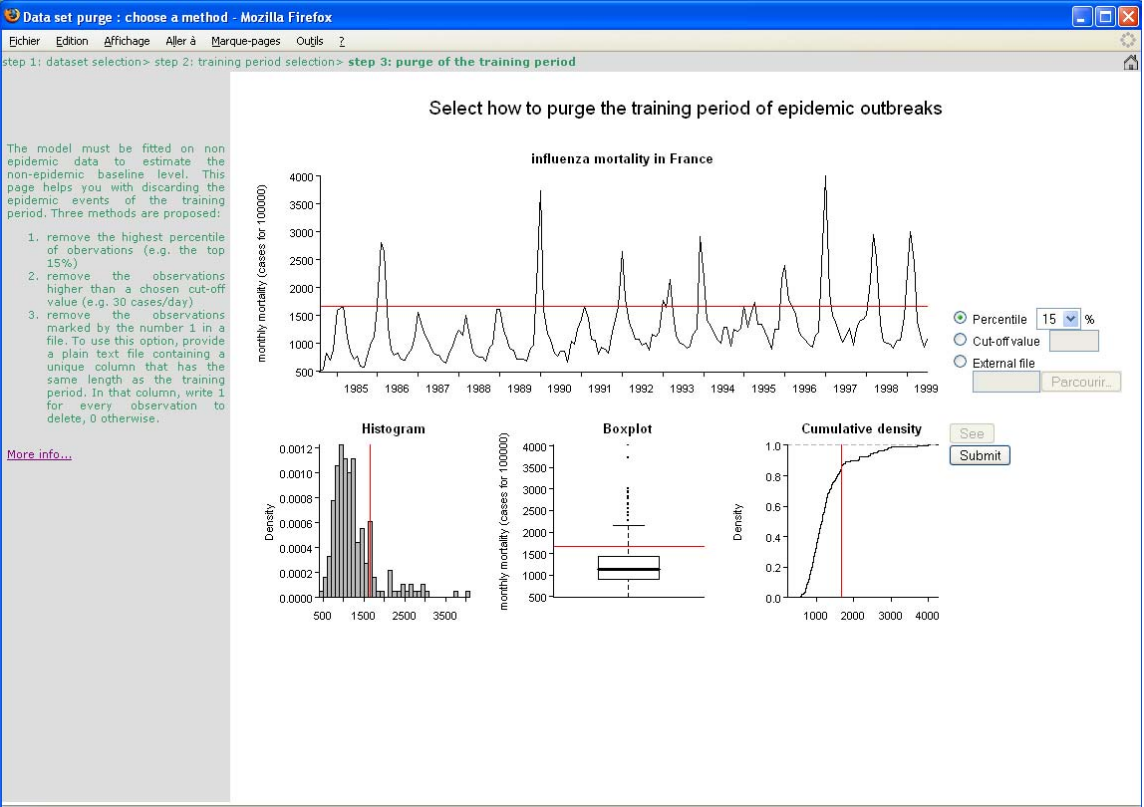
**Purge of the training period**. Interactive selection of the method used to purge the training period of past epidemic outbreaks. Option 1 (delete the highest percentile of observations) was chosen. The percentile was set to 15% in a scrolling list ranging 0% to 60%.

The second page allows excluding observations from the training period (Figure 2). Three options for excluding data are proposed. The user may select the upper percentile between 0% and 60% above which all data are excluded. Excluding all observations greater than a specified cut-off value is the second option. In the third option, the user provides a file of the same length as the training period flagging the observations as "keep" (value 0) or "exclude" (value 1). To guide the percentile or cut-off selection, histograms and cumulated density plot are provided. In Figure 2, the user selected to exclude all observations greater than the 15% upper percentile.

The third page allows the user to select the mathematical form for the baseline model. This page is dependent on the type of analysis, prospective *or *retrospective. For the retrospective analysis, nine models are available, combining the three choices for the trend and periodicity (see Table 2). Using the automated selection feature, the model with cubic trend and annual periodicity is chosen for baseline P&I mortality. Figure 1 presents the detail of the selection algorithm.

In the third page, the user defines the epidemic threshold by selecting a percentile of the prediction distribution, between 50% and 100%. Here, default value (95%) was selected. Increasing this value will lead to less observations outside the thresholds and more specific detection. On the contrary, decreasing the threshold will increase sensitivity and timeliness of the alerts.

To avoid making alerts for isolated data points, a minimum duration above the threshold may be required. Default values are 14 days (2 weeks) for daily and weekly data, and 1 month if the data are monthly-aggregated. The beginning of the epidemic is the first date the observations exceed the threshold, and the end the first time observations return below the threshold. Here, the default value (1 month) was selected.

**Figure 3:**
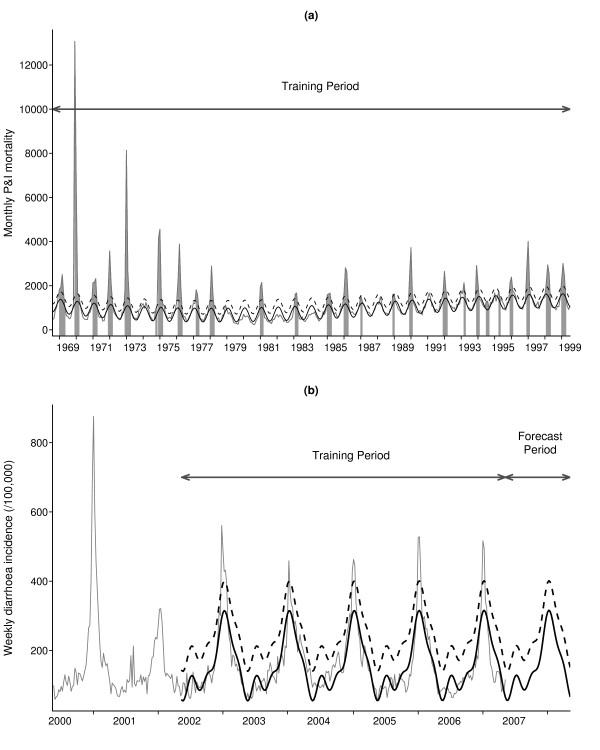
**Graphical output of the software**. (a) Retrospective detection of influenza epidemics from monthly P&I mortality in France, 1968–1999. (b) Prospective analysis of gastrointestinal disease (2002–2007) and model-based extrapolation for 2008 with epidemic threshold. In all graphs: observed (grey), model (black), upper forecast limit (dashed).

The application provides plots of the time series, the baseline level, the threshold and the detected epidemics (Figure 3a). A first table is output with the expected baseline and threshold values at each date in the dataset. A second table shows the dates and excess mortality for all detected epidemics (summarized in Table 2). The excess mortality is defined as the cumulated difference between observations and baseline over the entire epidemic period. Excess percentages are also provided, calculated as the observed size divided by the sum of expected values throughout each epidemic.

### Prospective surveillance of gastrointestinal diseases

In this analysis, the user wishes to define epidemic thresholds for prospective monitoring of diarrhoea. We briefly summarize the differences between this analysis and the retrospective case. As above, a time series must be first provided (here we selected diarrhoea with weekly observations). After choosing "prospective analysis", the user must select the duration of the training period, typically a few years. We select five years for the training data. Data exclusion before fitting the baseline model is carried out as in the first example. The regression equation is limited to a linear trend, but all three periodicities are available. Here, the automated selection leads to a model with a linear trend, and annual, semi-annual and quarterly periodic terms.

Alert thresholds are defined by selecting a percentile of the prediction distribution, between 50% and 100%. A typical choice is 95%. The application then generates a plot showing the whole data, the baseline and threshold values over the training period and model extrapolation for the following year (Figure 3b). An output table contains the expected baseline and threshold values for each date in the dataset and the following year.

## Discussion

We have presented an online application for analysing epidemiologic surveillance time series. The program can be used to extract the dates and size of past epidemics, or to establish epidemic thresholds for prospective surveillance. We intend this application to be a practical tool for field and public-health practitioners. We designed a user-friendly interface that provides default-values options and interactive graphical feedback. Since all the parameters can be changed by the user, the program provides an easy way to check how the analysis changes with different choices.

The epidemiologic time series most suitable for analysis are those where the monitored signal consists of a seasonal background with outbreaks. This is clearly the case for influenza surveillance data. Influenza-like syndromes occur at all times of the year, although typically more in the winter than the summer, even when no influenza viral strain is circulating. Viral testing is considered the gold standard method to provide the real number of influenza-affected patients but since this test is not part of routine diagnoses, morbidity and mortality in a population can not be specifically attributed to influenza. One way to estimate the impact of influenza in a population from surveillance data including surveillance of influenza-like syndromes, pneumonia or influenza associated admissions, or cause-specific mortality, is to use statistical methods such as periodic regression. This hypothesis also holds for other infectious diseases, for example gastroenteritis where syndromes under surveillance (diarrhoea, fever) can be due to various pathogens which are more active in some seasons than others. Alternative detection methods exist that do not rely on the hypothesis of a seasonal baseline. For instance, Hidden Markov Models assume that the observations are generated from a finite mixture of distributions governed by an underlying Markov chain [25,26]. These methods have shown good aptitude in distinguishing epidemic and non epidemic phases in seasonal and non-seasonal time series. Another alternative is control-chart methods, which may be calibrated on data from recent months rather from previous years [27].

A minimum of one year historical data is required to fit the models discussed here, but we note that more reliable predictions require at least two or three year historical data to calculate the baseline level. Other methods have been developed for disease surveillance with limited historical data sets [27,28]. We also recommend, for the prospective setting, to make sure that the one year long predictions begin outside the epidemic season, in order to highlight the incoming epidemic in its entirety. While first and second degree polynomial trends are frequently used in periodic regression models in the literature [2,3], we have added the option of a third degree polynomial to offer more flexibility, only for the retrospective analysis. For the seasonal components, we included the most widely used periodicities, ie 12, 6 and 3 months. We did not propose higher degree polynomials or seasonal terms because higher order terms may be more prone to result in unidentifiable models or other problems with model fit.

The application is based on a general periodic regression model that contains most previous published models as special cases. Yet, we did not implement some specialised models encountered in the literature. For example, some authors modelled the secular trend with a smoothing spline fitted on summer months [12,29]. Others included autoregressive terms in their models [5,30,31]. Additional variables may also be incorporated into the regression model, for example day of the week, holiday, and post-holiday effects [7], sex and age [32], or temperature and humidity [5]. A few authors replaced the epidemic values in the training period by expected non-epidemic values, rather than deleting them [10,33]. We have not included these options in the application for reasons of parsimony. One of the most important features of an online tool such as the one presented here is that it should allow inferences to be made by front-line practitioners who often do not have detailed knowledge of statistical software. We have attempted to balance the desire to provide a user-friendly interface while at the same time offering sufficient options to cover the needs of most surveillance datasets.

## Conclusion

The online application presented here should be a valuable tool for public health surveillance. Its user-friendly interface facilitates fairly complex modelling, offering public health practitioners the possibility to rapidly investigate the burden of epidemics, or to utilise the same statistical approaches to set epidemic thresholds for prospective surveillance.

## Availability and requirements

• **Project name: **Periodic regression models

• **Project home page: **

• **Operating systems: **Web based application

• **Programming language: **R, PHP, Javascript

• **Other requirements: **Javascript supported and activated on the web browser (tested with Mozilla 5.0 and Internet Explorer 7.0).

## Competing interests

The author(s) declare that they have no competing interests.

## Authors' contributions

CP designed and programmed the application and drafted the manuscript. PYB helped to conceive the application and to draft the manuscript. BJC helped with the program and with drafting the manuscript. FC participated in the program design. AF helped to draft the manuscript. SA helped designing the application. AJV participated in designing the application and drafting the manuscript. All authors have read and approved the final manuscript.

## Pre-publication history

The pre-publication history for this paper can be accessed here:



## Supplementary Material

Additional file 1 **R codes**. The zip file contains all the R codes used in the web site and an instruction file. You can directly run the scripts with the R software, following the directions in the instruction file, to obtain the graphical outputs and the tables. Click here for file
